# Aharonov-Bohm Interference and Phase-Coherent Surface-State Transport in Topological Insulator Rings

**DOI:** 10.1021/acs.nanolett.3c00905

**Published:** 2023-07-03

**Authors:** Gerrit Behner, Abdur Rehman Jalil, Dennis Heffels, Jonas Kölzer, Kristof Moors, Jonas Mertens, Erik Zimmermann, Gregor Mussler, Peter Schüffelgen, Hans Lüth, Detlev Grützmacher, Thomas Schäpers

**Affiliations:** † Peter Grünberg Institut (PGI-9), Forschungszentrum Jülich, 52425 Jülich, Germany; ‡ JARA-Fundamentals of Future Information Technology, Jülich-Aachen Research Alliance, Forschungszentrum Jülich and RWTH Aachen University, 52425 Jülich, Germany

**Keywords:** Topological insulators, ring interferometer, Aharonov-Bohm effect, topological surface states, ballistic transport, phase-coherent transport

## Abstract

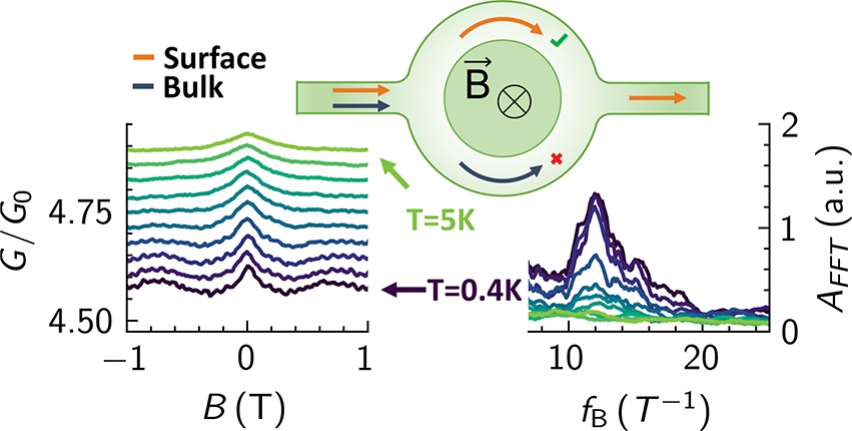

We present low-temperature magnetotransport measurements on selectively grown Sb_2_Te_3_-based topological insulator ring structures. These devices display clear Aharonov-Bohm oscillations in the conductance originating from phase-coherent transport around the ring. The temperature dependence of the oscillation amplitude indicates that the Aharonov-Bohm oscillations originate from ballistic transport along the ring arms. We attribute these oscillations to the topological surface states. Further insight into the phase coherence is gained by comparing with similar Aharonov-Bohm-type oscillations in topological insulator nanoribbons exposed to an axial magnetic field. Here, quasi-ballistic phase-coherent transport is confirmed for closed-loop topological surface states in the transverse direction enclosing the nanoribbon. In contrast, the appearance of universal conductance fluctuations indicates phase-coherent transport in the diffusive regime, which is attributed to bulk carrier transport. Thus, it appears that even in the presence of diffusive *p*-type charge carriers in Aharonov-Bohm ring structures, phase-coherent quasi-ballistic transport of topological surface states is maintained over long distances.

Phase-coherence has a great impact on transport in mesoscopic systems, which leads to many interesting effects, visible through their quantum mechanical correction to the conduction as a function of magnetic field or gate voltage.^1,2^ Typical phenomena associated with phase-coherent transport are weak (anti)localization, universal conductance fluctuations (UCFs), or Aharonov-Bohm (AB) oscillations.^1^ Recently, phase-coherent transport has also been studied in three-dimensional topological insulators (TIs) such as Bi_2_Te_3_, Sb_2_Te_3_, Bi_2_Se_3_, or their alloys, in which topologically protected spin-momentum locked surface states are present.^3,4^ Interest in these materials stems from applications in topoelectronic circuits and topological quantum computer architectures.^5−39^

In previous studies, various transport properties of straight three-dimensional TI-based nanowires and nanoribbons have been investigated theoretically and experimentally in micrometer- and nanometer-sized systems. The observed effects range from weak antilocalization and conductance fluctuations to the manifestation of quasi-ballistic transport of topologically protected surface states, inducing Aharonov-Bohm-type conductance oscillations when applying a magnetic field along the wire or ribbon.^9−16^

Three-dimensional TIs tend to be intrinsically doped due to the formation of crystal defects during growth, leading to an additional bulk transport channel as the Fermi level either crosses the conduction or valence band.^17,18^ With respect to phase-coherent transport, it is a difficult task to disentangle the contributions from the bulk and topologically protected surface states. On the one hand, the picture regarding UCFs is not so clear. In nanoribbons of ternary materials with a relatively small bulk contribution, the UCFs have been attributed to surface states,^14^ while in Bi_2_Se_3_ nanoribbons, they have been assigned to bulk carriers.^19^ On the other hand, the AB-type oscillations observed in nanoribbons are generally believed to be due to phase-coherent loops formed by topologically protected surface states in the transverse direction around the perimeter of the cross section.^9−13,15,16,20^ From the exponential decrease of the oscillation amplitude with temperature, it was deduced that the transport is quasi-ballistic.^11,15,16^ However, little information has been available on the phase-coherent transport of topologically protected surface states along the axial direction of the nanoribbon, i.e., the direction along which the (surface-state) current flows. To address this issue, we measure planar Sb_2_Te_3_ ring-shaped interferometers and investigate the Aharonov-Bohm effect with an out-of-plane magnetic field. Here, we identify a clear peak in the Fourier spectrum of the magnetoconductance corresponding to magnetic flux quantum-periodic oscillations. From the decrease of that peak with the temperature, the corresponding transport regime is identified. The comparison of different ring sizes as well as a detailed analysis of the spectrum of the UCFs and the Aharonov-Bohm oscillations in straight nanoribbons allows a comprehensive investigation of the phase-coherence in the different transport channels. The interpretation of the transport measurement data is supported by quantum transport simulations.

The 20 nm-thick Sb_2_Te_3_ ring structure was grown by molecular beam epitaxy employing a selective-area growth approach using a prepatterned SiO_2_/Si_3_N_4_-covered Si(111) substrate (for details, see Supplementary Note 1).^21^ To prevent oxidation, the Sb_2_Te_3_ layer was capped by a 5 nm-thick AlO_*x*_ layer. For ohmic contacts, a 20 nm-thick Ti layer and a 70 nm-thick Pt layer was used. From measurements on a 500 nm-wide Hall bar at 1.5 K, we determined a hole carrier concentration of 7.4 × 10^13^ cm^–2^ and a mobility of 152 cm^2^/(V s) (see Supplementary Note 2). Rings of two different sizes were investigated, i.e., samples A and B with an outer radius of 150 and 200 nm, respectively, both with an annulus width of 50 nm. Figure 1 shows a schematic of a selectively grown ring structure as well as a scanning electron micrograph of sample A. To further characterize the properties of the Sb_2_Te_3_ layers, a 100 nm-wide nanoribbon (sample C) prepared in the same run as the ring structures was fabricated. The conductance of the ring structures was measured in a ^3^He-cryostat with a base temperature of 400 mK, while the nanoribbon was measured in a variable temperature insert with a base temperature of 1.4 K. The magnetic field *B* is applied perpendicularly to the substrate plane.

**Figure 1:**
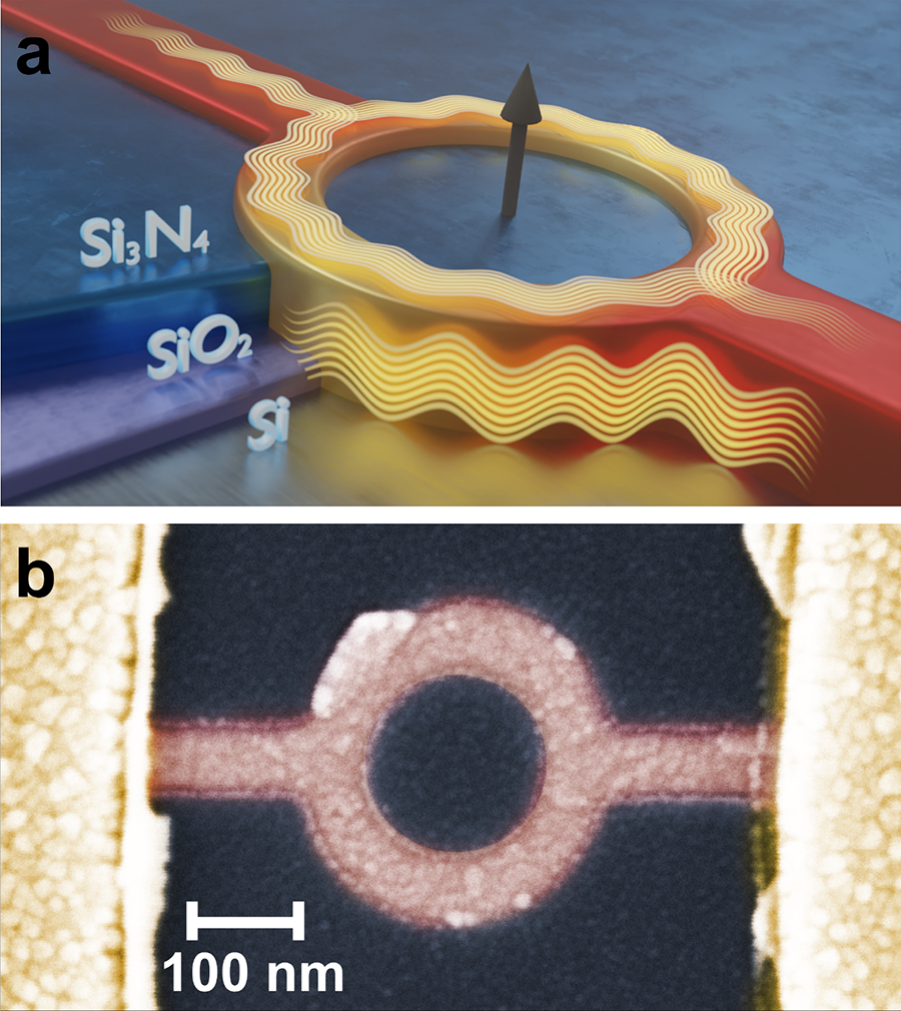
Aharonov-Bohm interferometer. (a) Schematics of the selectively grown ring structure. (b) Scanning electron micrograph of a selectively grown Sb_2_Te_3_-based ring device (sample A) with an inner and outer radius of 100 and 150 nm, respectively.

Figure 2a shows the normalized magnetoconductance *G*/*G*_0_ of a ring interferometer structure (sample A) with *G*_0_ = 2*e*^2^/*h*. The measurement temperature is varied from 0.4 to 5.0 K. The magnetic field is oriented perpendicularly to the substrate plane so that a magnetic flux is threading the ring aperture. The corresponding data of the second ring structure (sample B) are presented in Supplementary Note 2.

**Figure 2:**
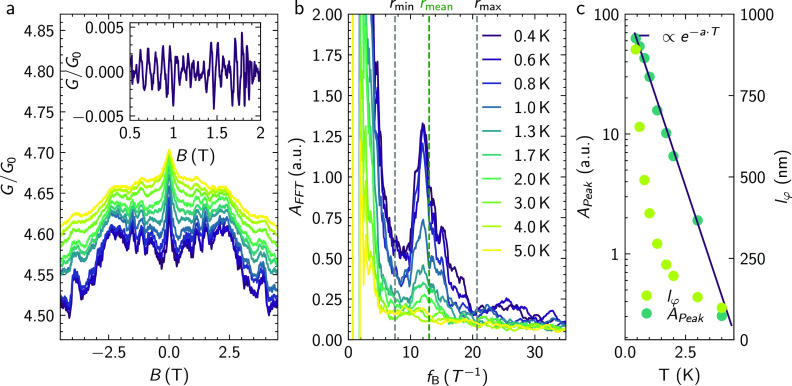
Magnetotransport of a topological insulator ring structure. (a) Normalized magnetoconductance of sample A at temperatures in the range of 0.4 to 5.0 K, with *G*_0_ = 2*e*^2^/*h*. The inset shows a detail of the oscillations at 800 mK in a smaller magnetic field range. The measured period *ΔB* of 82.5 mT fits accurately to the peak observed in the FFT of the measured data. The legend for the different temperatures is given in (b). (b) Fourier spectrum of the magnetoconductance shown in (a). The expected frequency according to the minimum and maximum radius as well as the mean radius are indicated by dashed lines. (c) Integrated amplitude  of the peak in the FFT at 12T^–1^ as well as the phase coherence length *l*_φ_ as a function of temperature. The solid line represents an exponential fit.

The magnetoconductance exhibits several features. The most striking one is a peak at zero magnetic field, which is due to weak antilocalization. This peak structure has been observed previously in TI nanoribbon structures of similar width and is due to electron interference combined with strong spin–orbit coupling (see Supplementary Note 3).^14,15^ Another feature is pronounced conductance fluctuations with with an amplitude on the order of 0.05 G_0_ and an oscillation period of multiple Tesla, in particular at low temperatures. These are caused by the interference of a limited number of trajectories due to the small dimensions of the sample,^22^ which will be discussed in more detail at a later stage to provide complementary information on phase-coherence. A closer look at the magnetoconductance reveals that regular oscillations with smaller amplitudes are superimposed on the conductance fluctuations. A magnification of a smaller magnetic field region is shown in the inset of Figure 2a. We found that the oscillation period is about *ΔB* = 82.5 mT. We attribute these regular features to the Aharonov-Bohm effect in the ring-shaped conductor.^23^ The small variation of the oscillation period in the inset of Figure 2a is the result of a distribution of all possible paths throughout the ring. Indeed, the period *ΔB* fits very well to oscillations with a flux period of ϕ_0_ = *ΔB* × *A*, with ϕ_0_ = *h*/*e* the magnetic flux quantum and *A* = *πr*_mean_^2^ the area of the disc with radius equal to the mean radius of the ring: *r*_mean_ = 125 nm.

The periodic features in the magnetoconductance are analyzed by using a fast Fourier transform (FFT), as shown in Figure 2b. Note that the Fourier transform was applied to the original data without any filtering. The FFT shows a distinct peak at a frequency *f*_*B*_ of 12T^–1^, which corresponds to the expected value for the mean radius of the ring, indicated by the vertical dashed green line in Figure 2b. In general, the peak lies within the frequency limits given by the inner (*r*_min_) and outer (*r*_max_) ring radii, indicating that the trajectories of the electron partial waves cover the entire ring area. As the temperature increases, the height of the peak decreases, corresponding to a reduction of the oscillation amplitude. At about 3.0 K, the peak has disappeared. In addition to the peak at about 12 T^–1^, a weaker feature is observed at about 25 T^–1^, where the second harmonic is expected.

From the decrease of the integrated peak height  at 12 T^–1^ in the FFT with increasing temperature, we estimate the phase-coherence length *l*_φ_. The integration is performed within a window bounded by the frequencies corresponding to flux quantum periodicity when considering the inner and outer radius of the ring (Figure 2b). We consider an exponential decay ,^24^ with *πr*_mean_ = 393 nm being the length of one of the ring arms and  being a measure of the oscillation amplitude. Indeed, the peak height decay is fitted very well by an exponential decrease with *l*_φ_(*T*) ≈ *T*^–1^, as shown in Figure 2c. Due to a strongly fluctuating background, a reference value for Δ*G* cannot be extracted reliably from the raw data. The amplitude of the peak in the Fourier transformed data is therefore assumed to be proportional to the change in conductance *ΔG* ∝ *A*_Peak_. From the fit, we obtain a phase-coherence length of *l*_φ_ = 891 nm at a temperature of 0.4 K, which is considerably longer than the length of the ring arm. Figure 2c also displays the temperature dependence of phase-coherence length *l*_φ_. It shows a clear power dependence as a function of temperature. The dominating phase-breaking mechanisms in similar films have been determined to arise from Niquist electron–electron interaction for disordered systems.^25^

Aharonov-Bohm oscillations arise when the phase-coherence length *l*_φ_ is on the order of the length of a ring arm, which is the case for our ring, as shown above. In principle, in our intrinsically doped TI samples, the oscillations can originate from bulk carriers as well as from charge carriers in topologically protected surface states. However, the exponential decay of the FFT amplitude with *l*_φ_(*T*) ≈ *T*^–1^ indicates that the transport is in the quasi-ballistic mesoscopic regime.^26,27^ Hence, we anticipate that the observed AB oscillations are mainly due to the transport of topologically protected surface states. Indeed, in TI nanoribbons it was deduced that these surface states with spin-momentum locking have an enhanced transport mean free path due to strongly anisotropic scattering.^19,28^ The presence of a high-mobility surface channel is also consistent with Shubnikov-de Haas measurements on Sb_2_Te_3_ layers.^29^ Here, it was also found that the surface-state carrier concentration is about 1 order of magnitude lower than the total one. Additional information on the relevant transport regime can be found in the Supplementary Note 2. Note that the oscillation amplitude in Figure 2 is small compared with the conductance quantum. This can be due to a number of reasons. The resistances in series from the leads and contacts as a result of the two-probe measurement reduce the overall transmission and would therefore also reduce the absolute amplitude of the oscillation. A suppressed amplitude can also be present due to disorder interaction, which is supported by the results of the simulations presented in Figure 3.

**Figure 3:**
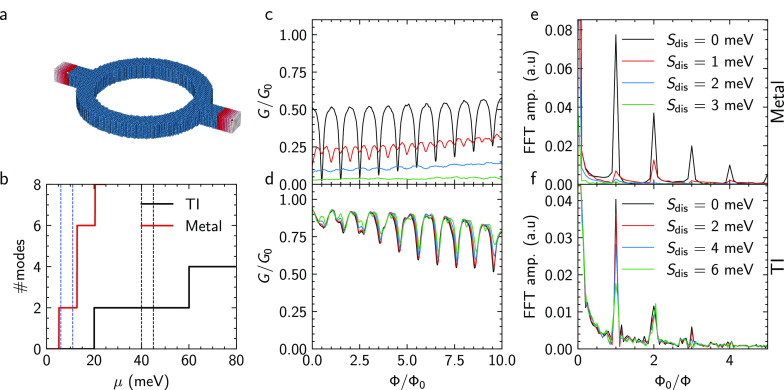
Tight-binding simulations of a metallic and topological insulator ring structure. (a) Schematic of the tight-binding model, with semi-infinite leads (a few unit cells indicated in red) attached to a (disordered) scattering region (in blue). (b) The number of transport channels as a function of energy for a bulk metallic ring and a (bulk-insulating) TI ring. The vertical dashed lines delineate the energy window over which the magnetoconductance is averaged. (c, d) The conductance as a function of the magnetic flux threading the aperture of (c) a bulk metallic ring and (d) a (bulk-insulating) TI ring for different values of the disorder strength in the scattering region. (e, f) Fourier transform of the conductance profile in (c) and (d), respectively.

To better understand the impact of elastic scattering due to disorder on the AB effect of the ring for bulk versus topological surface states, we performed quantum transport simulations. We make use of the quantum transport simulation package Kwant,^30^ the efficient parallel sparse direct solver MUMPS,^31^ and the Adaptive package^32^ to efficiently sample the parameter space, i.e., energy and flux. We employ the same tight-binding modeling approach as in refs (33 and 34) and refer to these works for more details. Disorder is considered by adding a randomly fluctuating on-site energy with a characteristic disorder strength, *S*_dis_, in the scattering region of the tight-binding model. In Figure 3, the simulation results are summarized. The assumed sample geometry for the simulation is depicted in Figure 3a. We compare the magnetoconductance of a bulk metallic ring with that of a bulk-insulating TI, where the bulk metallic states are described as free electron gases with an effective mass that is appropriate for the bulk states of Sb_2_Te_3_ near the Fermi level. The energy window is chosen for both systems such that they have a comparable magnetoconductance in the clean limit without disorder (Figure 3b). As can be seen in Figure 3c, without any disorder, the metallic ring displays the most pronounced AB effect, with the appearance of many higher harmonics (cf. Figure 3e). When disorder is introduced, however, the magnetoconductance oscillations are quickly suppressed as well as the conductance itself. The bulk metallic states are easily driven into a highly diffusive regime by disorder, which hinders transport along the ring and the corresponding AB signature. For the TI surface states, the behavior is quite different (cf. Figures 3d and f). While the AB peak and its harmonics in the Fourier spectrum are not so pronounced as for the metallic ring in the clean limit, disorder has a much weaker impact on the conductance and its flux quantum-periodic oscillations. This reflects the resilience of TI against elastic backscattering in the presence of disorder, which is also observed in straight nanoribbons and multiterminal junctions.^11,15,34−36^ Because of their spin-momentum locking properties and being bound to the surface, a robust (quasi)ballistic transport regime can be established for TI surface states even in the presence of relatively strong disorder throughout the ring geometry. In combination with the analysis presented in the following section on the presence of a second transport channel with diffusive properties, the results of the theoretical analysis allow us to attribute the quasibalistic contributions to transport in the form of Aharonov-Bohm oscillations to the presence of disorder resilient surface states in the material.

In order to gain more information on the phase-coherent transport, the investigation of Aharonov-Bohm oscillations is followed by an analysis of the conductance fluctuations also present in the magnetoconductance shown in Figure 2a. The temperature dependence of *l*_φ_ relevant for this phenomena can be determined from the correlation field *B*_c_. This quantity is extracted from the normalized fluctuation patterns in magnetoconductance *δG*/*G*_0_ depicted in Figure 4a obtained after subtracting the slowly varying background and filtering out the Aharonov-Bohm oscillations.

**Figure 4:**
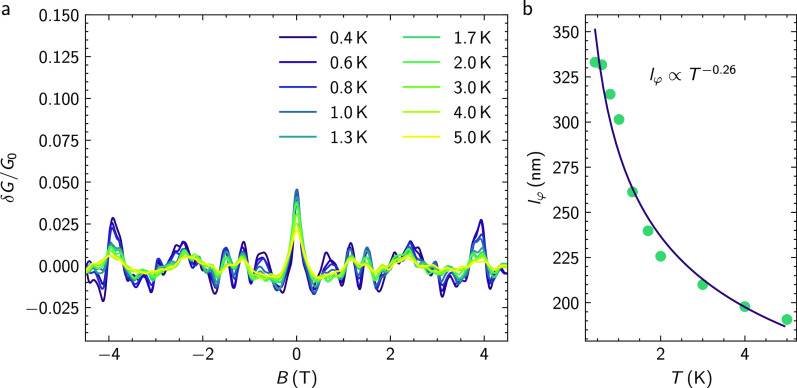
Universal conductance fluctuations. (a) Normalized fluctuation pattern present in the magnetoconductance measurements of the Sb_2_Te_3_ ring structure presented earlier with *G*_0_ = 2*e*^2^/*h*. The magnetic field is oriented perpendicular to the ring structure. (b) Phase-coherence length *l*_φ_ of the ring structure extracted from the correlation field *B*_c_ as a function of temperature. The blue line indicates an exponential decrease in the phase-coherence length following *T*^–0.26^.

It can clearly be seen that the fluctuation amplitude substantially decreases as temperature increases, while the pattern itself is consistent over all temperatures. The correlation field *B*_c_ is determined using the autocorrelation function: *F*(*ΔB*) = ⟨*δG*(*B* + *ΔB*)*δG*(*B*)⟩.^22^ Here, the full-width at half-maximum *F*(*B*_c_) = 1/2*F*(0) defines *B*_c_. In the diffusive regime, *l*_φ_ can be determined using *l*_φ_ ≈ *γϕ*_0_/*B*_c_*d*,^37^ with *d* the width of the ring arms and the width of leads to the ring, which is 50 nm in our case. For the prefactor γ, we choose 0.42 for *l*_φ_ larger than the thermal length (see Supplementary Note 2).^37^ The resulting values of *l*_φ_ determined from the correlation field are listed in Figure 4b. As indicated by the blue line, the decrease of the phase-coherence length *l*_φ_ with temperature increase can be fitted by a dependency of *l*_φ_ ∝ *T*^–0.26^. The temperature dependence of the sample is slightly lower than the expected dependence of *T*^–1/3^ for a diffusive quasi one-dimensional system.^38^ The maximum of *l*_φ_ = 330 nm is smaller than the corresponding value determined from the Aharonov-Bohm oscillations. We attribute this discrepancy to different contributions to the overall phase-coherent transport. As outlined in the previous section, we concluded that the Aharonov-Bohm oscillations originate from (quasi)ballistic transport of topologically protected surface states. In contrast, conductance fluctuations by nature show up only in the diffusive transport regime. We can therefore attribute the appearance of conductance fluctuations to diffusive bulk transport.

For the topological surface states of TI nanoribbons, Aharonov-Bohm-type oscillations are also expected to show up with the application of an axial magnetic field. In this case, the oscillations originate from the interference of topologically protected surface states, enclosing the magnetic flux penetrating the cross section of the nanoribbon. We verify this effect as well by measuring a 100 nm-wide Sb_2_Te_3_ nanoribbon from the same growth run as the planar ring under application of an in-plane field along the nanoribbon axis. Figure 5a shows the corresponding magnetoconductance measurements at temperatures in the range of 1.7 K to 20 K under application of a magnetic field up to ±13T. The data show a peak at zero magnetic field, which can be attributed to weak antilocalization.^14,15^ On top of this feature, clear low-frequency magnetic field-dependent oscillations are observed. The corresponding curves after background subtraction are depicted in Figure 5b. A clear peak at a frequency of around 0.45 T^–1^ can be seen in the Fourier spectrum in Figure 5c. The cross-sectional area determined from the frequency is 1.86 × 10^–15^ m^2^, which matches very well to the cross-sectional area of 2 × 10^–15^ m^2^, which is determined from the film thickness of 20 nm at a nanoribbon width of 100 nm. Note that the absence of *h*/2*e* periodic Aronov-Altshuler-Spivak (AAS) oscillations can be attributed to the absence of backscattering in spin-momentum locked surface states. Furthermore, the presence of single peak in the FFT spectrum excludes contributions of coherent loops of various sizes in the bulk channel. The temperature dependence of the peak suggests a strong dependence on the phase-coherence of our carriers. The peak amplitude should vanish with temperature corresponding to a vanishing phase-coherence length of a carrier as a function of temperature. Similarly to the planar ring structures, we determined the decay of the Aharonov-Bohm integrated peak height in the FFT spectrum shown in Figure 5b with temperature. Once again, the decay follows an exponential dependence according to , with *P* the nanoribbon perimeter. The temperature dependence indicates that the transport is quasi-ballistic.^11,15,16^ From the fit, we deduced a phase-coherence length of *l*_φ_ = 600 nm at 2 K. The observed oscillations can be attributed to phase-coherent oscillations around the perimeter of the nanoribbon. This effectively proves the existence of surface states in the investigated material.

**Figure 5:**
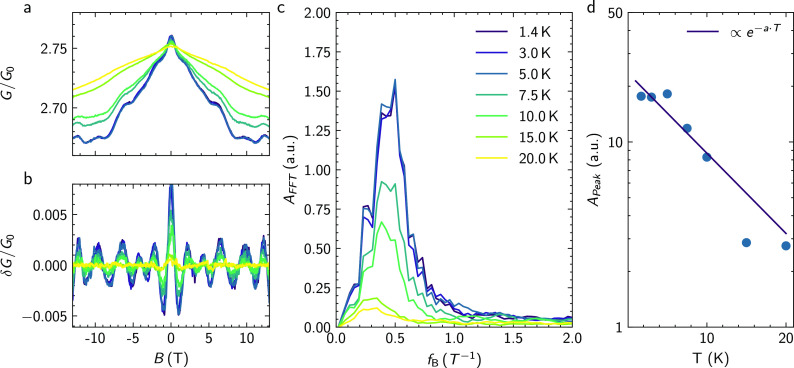
Magnetotransport in a nanoribbon. (a) Magnetoconductance of a nanoribbon structure (sample C) under application of an axial in-plane magnetic field in the range of 1.4 K to 20 K. The legend can be found in (c). (b) Corresponding magnetoconductance after subtracting the slowly varying background signal using a Savitzky-Golay filter. (c) Fourier transform of the magnetoconductance data shown in (b) at different temperatures. (d) Amplitude of the peak in the FFT spectrum as a function of temperature. The solid line represents the fit.

In conclusion, from the temperature dependence of the Aharonov-Bohm oscillation amplitude Sb_2_Te_3_ ring interferometers, we found that the phase-coherent transport takes place in the quasi-ballistic regime. By comparing the quantum transport simulations of a metallic and topological insulator ring structures, we conclude that the quasi-ballistic transport can be attributed to the topologically protected surface states. The underlying reason is that these states are resilient against elastic backscattering in the presence of disorder, unlike the diffusive bulk states. In addition to the periodic Aharonov-Bohm oscillations, the magnetoconductance trace also contains irregular conductance oscillations. Since the appearance of this phenomena requires transport in the diffusive regime, we conclude that transport in the bulk channel is responsible in this case. Finally, on straight nanoribbons fabricated in the same growth run, regular Aharonov-Bohm oscillations are observed under the application of an axial magnetic field. As for the planar ring structures, a quasi-ballistic regime was identified. Our investigation on planar ring structures as well as on straight nanowire thus leads us to the conclusion that the phase-coherent transport in lateral as well as in transverse direction is quasi-ballistic. Furthermore, it seems that the transport in the topological surface states is decoupled from the phase-coherent diffusive transport in the bulk channel. The present work is an important milestone to the distinction between quantum transport in topologically protected surface states and in the bulk channel. Our results thus help to design future topological devices based on phase-coherent transport with topological insulator surface states.

## Supporting Information Available

Detailed description of sample preparation and measurement setup; Magneto-transport measurements on a Hall bar structure; Aharonov-Bohm oscillations measured on a second ring structure; Weak antilocalization analysis according to the Hikami-Larkin-Nagaoka formula (PDF)
