# Supplementary material for: Aharonov-Bohm Interference and Phase-Coherent Surface-State Transport in Topological Insulator Rings
Source: Nano Lett. 2023 Jul 3;23(14):6347–53. doi: 10.1021/acs.nanolett.3c00905 (PMC10375586; doi:10.1021/acs.nanolett.3c00905)
Supplement: Supplementary file 1 — nl3c00905_si_001.pdf [file nl3c00905_si_001.pdf]

Supporting Information: Aharonov-Bohm interference and phase-coherent surface-state transport in topological insulator rings

Gerrit Behner,[1,2,*] Abdur Rehman Jalil,[1,2] Dennis Heffels,[1,2] Jonas Kölzer,[1,2] Kristof Moors,[1,2] Jonas Mertens,[1,2] Erik Zimmermann,[1,2] Gregor Mussler,[1,2] Peter Schüffelgen,[1,2] Hans Lüth,[1,2] Detlev Grützmacher,[1,2] and Thomas Schäpers[1,2,†]

[1]*Peter Grünberg Institut (PGI-9), Forschungszentrum Jülich, 52425 Jülich, Germany*
[2]*JARA-Fundamentals of Future Information Technology, Jülich-Aachen Research Alliance, Forschungszentrum Jülich and RWTH Aachen University, Germany*

(Dated: June 1, 2023)

SUPPLEMENTARY NOTE 1: SAMPLE PREPARATION

The Sb_2Te_3 layer was grown by molecular beam epitaxy employing a selective-area growth approach [1]. For the substrate preparation, first, 5 nm of a Si(111) wafer was thermally converted into SiO_2. Subsequently, a 20-nm-thick Si_3N_4 layer was deposited by low-pressure chemical vapor deposition. The ring and nanoribbon structures were defined by electron beam lithography. Using reactive ion etching (CHF_3/O_2) and hydrofluoric acid wet etching, the Si_3N_4 and the SiO_2 layers were etched, respectively, to reveal the Si(111) surface locally. The structured Si_3N_4/SiO_2 layers formed the selective-area growth mask. The standard parameters for selective growth of Sb_2Te_3, given a substrate temperature of 300 °C, a Sb-cell temperature of 470°C, and a Te-cell temperature of 325 °C, resulted in a growth rate of 7 nm/h. The 20-nm-thick TI ring and nanoribbon structures were grown in the Te-overpressure regime. Oxidation of the Sb_2Te_3 layer is prevented by a 5-nm-thick AlO_x capping layer. The Ohmic contacts composed of a 20-nm-thick Ti layer and a 70-nm-thick Pt layer were deposited on top of the TI layer after removing the AlO_x capping in the contact areas by wet chemical etching and argon sputtering. Rings with an outer radius of 150 nm (sample A) and 200 nm (sample B) with an annulus width of 50 nm were measured. To further characterize the properties of the Sb_2Te_3 layers, a 100-nm-wide nanoribbon (sample C) prepared in the same run as the ring structures was fabricated. For basic characterization of the Sb_2Te_3 layer a 500-nm-wide Hall bar structure was measured (see Supplementary Note 2).

The measurement of the ring structures were conducted in a ^{3}He-cryostat with a base temperature of 400 mK. The magnetic field is applied perpendicularly to the substrate plane using a superconducting magnet. The magnetotransport of the nanoribbon and Hall bar were measured in a variable temperature insert with a base temperature of 1.4 K. The current was measured using a standard two-probe voltage sensing lock-in setup for all samples.

SUPPLEMENTARY NOTE 2: MEASUREMENTS ON HALL BAR STRUCTURES

Basic Hall measurements have been conducted on a 500-nm-wide Hall bar structure in order to obtain the electronic material properties of the Sb_2Te_3 topological insulator thin film. The Hall bar was selectively grown in the same growth run as the ring and nanoribbon structures. Supplementary Figure 1 shows an electron beam micrograph of the Hall bar sample. The topological insulator material is grown with a thickness of about 15 nm, which is thinner than for the ring and nanoribbon structures owing to the dependence of the growth rate on the window area in the growth mask. A standard lock-in amplifier setup and a variable temperature insert with a base temperature of 1.5 K were used for the measurements. In Supplementary Figure 2a and b the Hall resistance as well as the longitudinal magnetoresistance are shown. The system shows p-type behaviour. This is visible from the slope of the Hall resistance. The Hall resistance increases linearly indicating an effective single-channel transport. The sample exhibits a monotonic positive magnetoresistance as well as a weak antilocalization feature manifesting itself as a cusp-like dip in resistance around zero field [2]. A hole concentration of $n = 7.4 \times 10^{13}\,\mathrm{cm}^{-2}$ and a mobility of $\mu = 152\,\mathrm{cm}^2/\mathrm{Vs}$ were obtained from the Hall and longitudinal resistance measurements.

Taking the relatively large carrier concentration into account, we assume that most of the carriers are located in the bulk valence band. Indeed, we estimated the bulk carrier concentration to be a factor of more than ten

* g.behner@fz-juelich.de
† th.schaepers@fz-juelich.de

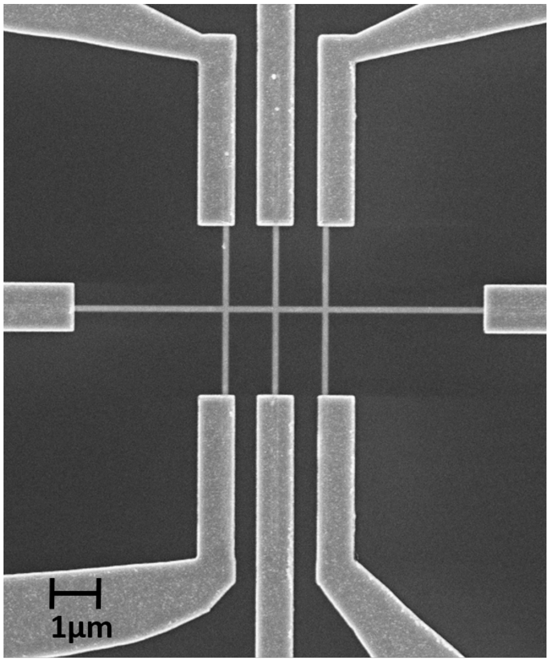

Supplementary Figure 1. Scanning electron micrograph of the 500-nm-wide Hall bar structure.

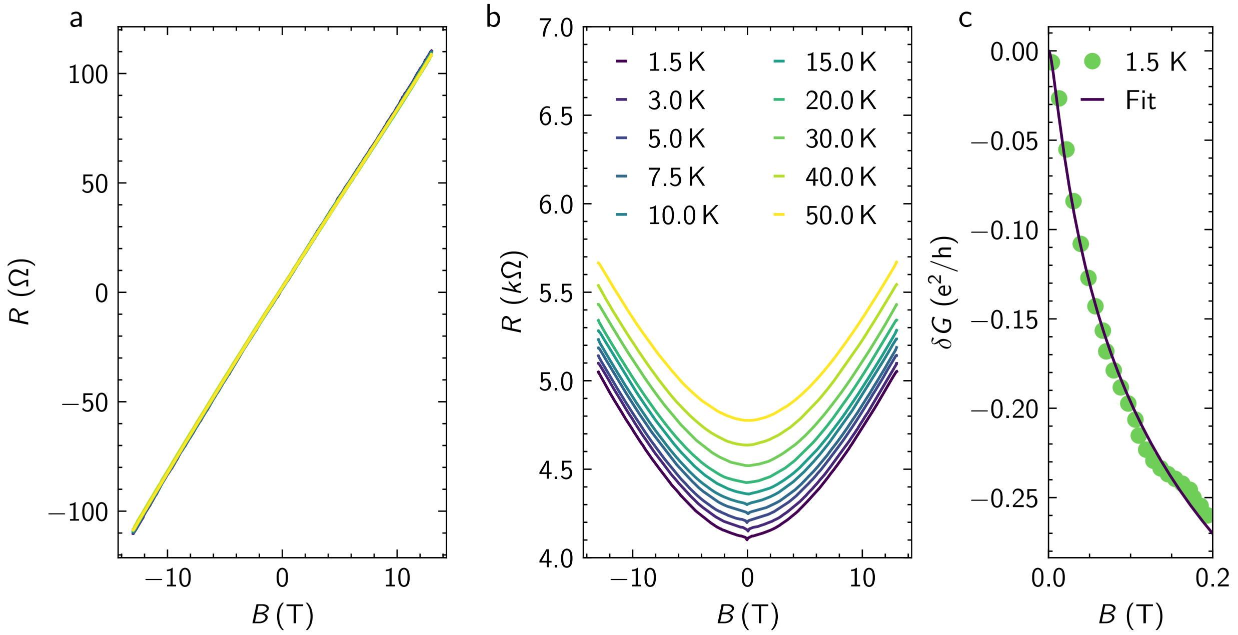

Supplementary Figure 2. a Hall resistance as a function of magnetic field for various temperatures of the Hall bar structure. b Corresponding longitudinal resistance as a function of magnetic field. c Measured magnetoconductance in a magnetic field range of 0 to 0.2 T and fit according to the Hikami-Larkin-Nagaoka theory.

larger than the concentration in the surface states. The dominating bulk contribution also results in an effective single-channel transport indicated by the linear increase of the Hall resistance shown in Supplementary Figure 2a. Thus, in order to determine the bulk transport parameters we neglect the contribution of the holes in the surface states. The approximate effect hole mass $m^* = m_0$, with m_e the free electron mass, was extracted from the band structure calculation of Zhang *et al.* [3]. Assuming that measured mobility is mainly governed by the bulk carriers we obtained an elastic scattering time of $\tau_e = (m^*\mu)/e$ of 4.8×10^{-14} s which together with an estimated Fermi velocity $v_{\rm F}$ of 1.3×10^5 m/s results in a diffusion constant of $\mathcal{D} = \frac{1}{3} v_{\rm F}^2 \tau_e \approx 0.41 \times 10^{-3}\,{\rm m^2/s}$ and an elastic mean

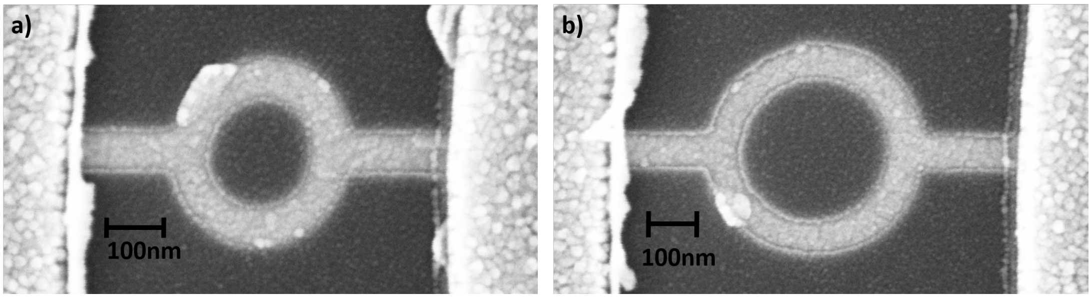

Supplementary Figure 3. a Scanning electron micro-graph of ring sample A in comparison to sample B which is shown in b.

free path $l_e = v_\mathrm{F}\tau_e \approx 11\,\mathrm{nm}$. The bulk Fermi velocity was calculated from $v_\mathrm{F} = (\hbar/m^*)(3\pi^2 n_\mathrm{3D})^{1/3}$, with n_3D the three-dimensional hole concentration. Having $\mathcal{D}$ available the thermal length defined by $l_T = \sqrt{\hbar\mathcal{D}/k_\mathrm{B}T}$ can be calculated. At a temperature of 1 K the thermal length is approximately 56 nm. Since the thermal length is in any case smaller than the phase-coherence length of bulk carriers determined from conductance fluctuations, we assumed a scaling factor of $\gamma = 0.42$ for the relation between the correlation field with l_φ in the main manuscript.

The phase-coherence length l_ϕ depends on the diffusion constant $\mathcal{D}$ via $l_\phi = \sqrt{\mathcal{D}\tau_\phi}$. Since each device consists of its own selectively-grown film, the transport properties and by that τ_e will be similar but not exactly the same. Thus, there might be a variation of l_ϕ when different devices are compared.

SUPPLEMENTARY NOTE 3: AHARONOV–BOHM OSCILLATIONS

We also investigate the magnetotransport of the second Aharonov-Bohm ring (sample B). For comparison, in Supplementary Figures 3a and b electron beam micrographs are shown for sample A and B, respectively. The outer radius of sample B is 200 nm, thus 50 nm larger than the one of sample A.

In Supplementary Figure 4a the normalized magnetoconductance G/G_0 of sample B is shown, with $G_0 = 2e^2/h$ and the temperatures varied between 0.4 K to 4.0 K. The magnetoconductance show the same kind of features as for sample A, i.e., a peak at zero resistance due to the weak antilocalization effect as well as conductance fluctuations. Superimposed, one also finds regular Aharonov-Bohm oscillations. This becomes clear by having a look on the magnification after background subtraction in a smaller magnetic field region depicted in Supplementary Figure 4a (inset). From that we extracted an oscillation period of $\Delta B = 47\,\mathrm{mT}$. The period ΔB fits very well to oscillations with a flux period of $\phi_0 = \Delta B \cdot A$, with $\phi_0 = h/e$ the magnetic flux quantum and $A = \pi r_\mathrm{mean}^2$ the ring area for the mean radius r_mean of 175 nm. The curves with subtracted background, i.e. Supplementary Figure 4a as well as Figures 3a and 5b (insets, main article) was created by subtracting a varying background from the original data in order to enhance the visibility of the oscillations. The reduced visibility is due to mainly the WAL and UCFs on top of the signal. The background trace is created using a Savitzky–Golay filter with an averaging window of 50 and 40 data points respectively, corresponding to a range of around 75 mT for the ring structure measurements. This is comparable to the oscillation period. Since the filtered data was not used for any further analysis and only for enhanced visibility this has no further impact on the validity of the analysis.

As for ring sample A, the periodic features in the magnetoconductance are analyzed by a fast Fourier transform (FFT), as shown in Supplementary Figure 4b. In the FFT spectrum we resolve a distinct peak at a frequency f_B of about $21.5\,\mathrm{T}^{-1}$, which fits to the expected value for the mean radius of the ring indicated by the vertical dashed green line in Supplementary Figure 4b. Increasing the temperature causes a decrease of the peak height. In contrast to sample A no second harmonic feature is observed in the Fourier spectrum.

The phase-coherence length l_φ was determined from the decrease of the integrated peak height $\mathcal{A}(T)$ at $21.5\,\mathrm{T}^{-1}$ in the Fourier spectrum with temperature. The integration is performed between frequencies corresponding to the minimum and maximum ring radii (cf. Supplementary Figure 4b). As for sample A, we assumed an exponential decay $\mathcal{A} \propto \exp(-\pi r_\mathrm{mean}/l_\varphi(T))$, with $\pi r_\mathrm{mean} = 550\,\mathrm{nm}$ the length of one of the ring arms. The peak height decay is very well fitted by an exponential decrease with $l_\varphi(T) \sim T^{-1}$, as shown in Supplementary Figure 4c. From the fit, we obtain a phase coherence length of $l_\varphi = 807\,\mathrm{nm}$ at a temperature of 0.4 K. The phase-coherence length l_{phi} is determined by comparing the fitted exponential decay of the peak amplitude $A_{peak} \propto a \cdot \exp(-b \cdot T)$ with the expected exponential decay of the conductance change of the AB- oscillation $\Delta G \propto \exp(-\pi \cdot r/l_\phi(T))$.

Supplementary Figure 4c also shows fits for the peak amplitude for different transport regimes. Generally the

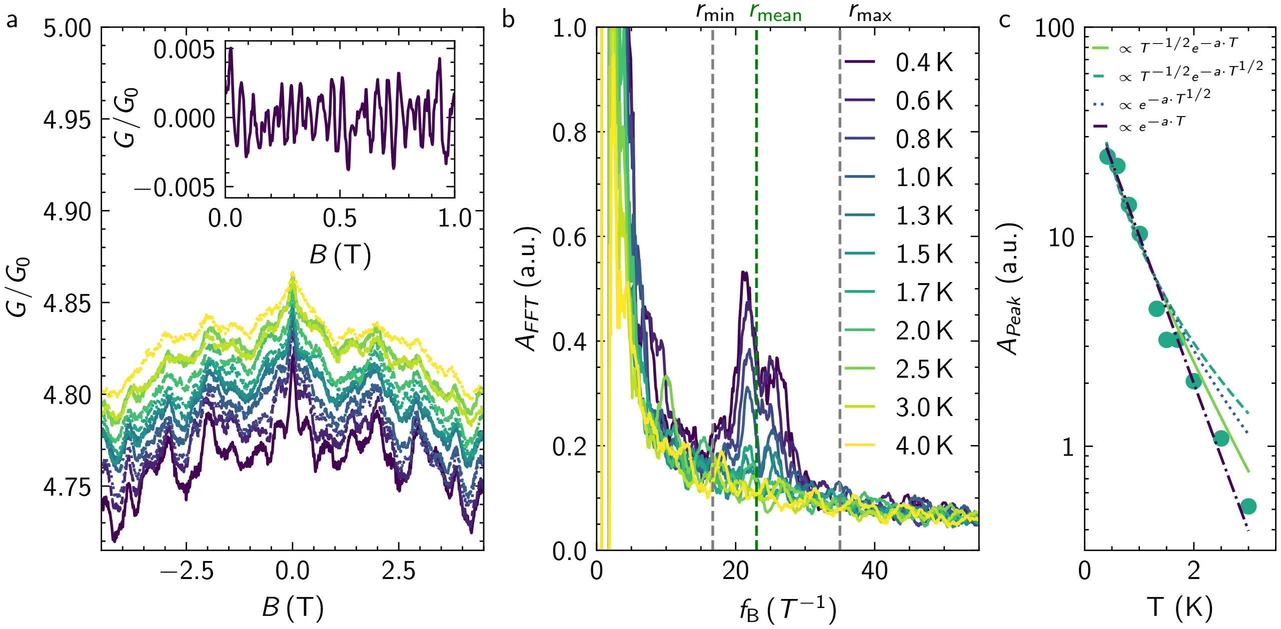

Supplementary Figure 4. Magnetotransport of ring structures: a Normalized magnetoconductance of sample B at temperatures in the range of 0.4 K to 4.0 K, with $G_0 = 2e^2/h$. The inset shows a detail of the oscillations at 400 mK in a smaller magnetic field range. The measured period ΔB of 47 mT, fits well to the peak observed in the FFT of the measured data. The legend for the curves belonging to different temperatures is given in b. b Fourier spectrum of the magnetoconductance shown in a. The frequency spectrum was smoothed with a moving average. The expected frequency according to the minimum and maximum radius as well as the mean radius are indicated by dashed lines. c Integrated amplitude $\mathcal{A}$ of the peak in the FFT at 23 T^{-1} as a function of temperature. The solid violet line represents an exponential fit with $\exp(-aT)$ corresponding to ballistic transport without thermal broadening. Also shown are fits assuming diffusive transport, i.e. with $\exp(-aT^{1/2})$, as well as thermal broadening, i.e. with prefactor $\sqrt{T}$.

temperature dependence of the Aharonov-Bohm oscillation amplitude can be described by [4, 5]

$$\delta G \propto \left(\frac{E_{\rm Th}}{k_{\rm B}T}\right)^{(1/2)} \exp(-\pi r/l_\varphi)\,, \tag{S1}$$

where $E_{\rm Th}$ is the Thouless energy [6], i.e., for diffusive conductors it is given by $E_{\rm Th} = \hbar\mathcal{D}/L^2$, with $\mathcal{D}$ the diffusion constant and L the ring circumference. For the ballistic case the situation for the Thouless energy is more subtle [7]. In case that the thermal energy $k_{\rm B}T$ is larger than $E_{\rm Th}$ thermal broadening occurs, leading to a pre-factor $T^{-1/2}$ of the oscillation amplitude. In case that $E_{\rm Th}$ is larger than $k_{\rm B}T$ this factor can be neglected. Furthermore, the temperature dependence of l_φ differs for the diffusive and ballistic case. Ludwig and Mirlin theoretically found that for the diffusive case the phase-coherence length is proportional to $T^{-1/2}$ [8], whereas in the ballistic case one expects $l_\varphi \sim T^{-1}$ [9]. In diffusive metallic and semiconducting rings a temperature dependence of $l_\varphi \sim T^{-p}$ with p in between 0.5 and 0.75 was observed [4, 10]. Whereas in clean semiconductor heterostructure rings [11, 12] as well as in clean graphene ring structures [13] a temperature dependence of $l_\varphi \sim T^{-1}$ was found, indicating a ballistic transport regime [9]. In topological insulator nanoribbons exposed to a magnetic field along the ribbon axis, Aharonov-Bohm oscillations are observed due to the presence of tubular topologically protected surface states. These systems were also found to be in the ballistic regime [14–16]. In Supplementary Figure 4c, fits assuming different scenarios are shown, i.e., thermal broadening vs. no thermal broadening and diffusive vs. ballistic transport. Obviously, a good fit was only obtained for the ballistic case without thermal broadening implying a large Thouless energy and a long mean free path. The latter can be explained by the reduced backscattering of carriers in topologically protected surface states.

SUPPLEMENTARY NOTE 4: WEAK ANTILOCALIZATION ANALYSIS

As mentioned in the previous section, the magnetoresistance shown in Supplementary Figures 2b exhibits a small cusp-like dip at zero field which can be assigned to the weak antilocalization effect. The weak antilocalization effect

for two-dimensional systems can be described by the the Hikami–Larkin–Nagaoka (HLN) formula [17]. A fitting to the experimental magnetoconductance (cf. Supplementary Figure 2c) results in a phase-coherence length of 225 nm with an α-factor of -0.37 at base temperature. The α factor is a measure of the number of transport channels and is close to the value of -0.5 corresponding to a single channel. Our values of l_φ and α indicate a strong bulk conduction of the system, which is typical for this type of samples as the charge carrier concentration is significantly higher and the mobility is lower than expected for Dirac surface states. The phase-coherence length extracted here fits well to the value obtained from the conductance fluctuations, indicating that both effects are governed by bulk carriers. The phase coherence length determined from the Aharonov-Bohm oscillations in ring structures is found to be considerably longer because in that case the transport takes place in a quasi-ballistic channel, i.e. topologically protected surface states.

SUPPLEMENTARY REFERENCES

[1] J. Kampmeier, C. Weyrich, M. Lanius, M. Schall, E. Neumann, G. Mussler, T. Schäpers, and D. Grützmacher, Selective area growth of Bi_2Te_3 and Sb_2Te_3 topological insulator thin films, Journal of Crystal Growth **443**, 38 (2016).

[2] C. Weyrich, T. Merzenich, J. Kampmeier, I. E. Batov, G. Mussler, J. Schubert, D. Grützmacher, and T. Schäpers, Magnetoresistance oscillations in MBE-grown Sb_2Te_3 thin films, Applied Physics Letters **110**, 092104 (2017).

[3] H. Zhang, C.-X. Liu, X.-L. Qi, X. Dai, Z. Fang, and S.-C. Zhang, Topological insulators in Bi_2Se_3, Bi_2Te_3 and Sb_2Te_3 with a single Dirac cone on the surface, Nature Physics **5**, 438 (2009).

[4] F. P. Milliken, S. Washburn, C. P. Umbach, R. B. Laibowitz, and R. A. Webb, Effect of partial phase coherence on aharonov-bohm oscillations in metal loops, Phys. Rev. B **36**, 4465 (1987).

[5] S. Washburn and R. A. Webb, Aharonov-Bohm effect in normal metal quantum coherence and transport, Advances in Physics **35**, 375 (1986).

[6] J. T. Edwards and D. J. Thouless, Numerical studies of localization in disordered systems, Journal of Physics C: Solid State Physics **5**, 807 (1972).

[7] A. Altland, Y. Gefen, and G. Montambaux, What is the Thouless energy for ballistic systems?, Phys. Rev. Lett. **76**, 1130 (1996).

[8] T. Ludwig and A. D. Mirlin, Interaction-induced dephasing of Aharonov-Bohm oscillations, Phys. Rev. B **69**, 193306 (2004).

[9] G. Seelig and M. Büttiker, Charge-fluctuation-induced dephasing in a gated mesoscopic interferometer, Phys. Rev. B **64**, 245313 (2001).

[10] J. Appenzeller, T. Schäpers, H. Hardtdegen, B. Lengeler, and H. Lüth, Aharonov-Bohm effect in quasi-one-dimensional $In_{0.77}Ga_{0.23}As/InP$ rings, Phys. Rev. B **51**, 4336 (1995).

[11] A. E. Hansen, A. Kristensen, S. Pedersen, C. B. Sørensen, and P. E. Lindelof, Mesoscopic decoherence in Aharonov-Bohm rings, Phys. Rev. B **64**, 045327 (2001).

[12] K.-T. Lin, Y. Lin, C. C. Chi, J. C. Chen, T. Ueda, and S. Komiyama, Temperature- and current-dependent dephasing in an Aharonov-Bohm ring, Phys. Rev. B **81**, 035312 (2010).

[13] J. Dauber, M. Oellers, F. Venn, A. Epping, K. Watanabe, T. Taniguchi, F. Hassler, and C. Stampfer, Aharonov-Bohm oscillations and magnetic focusing in ballistic graphene rings, Phys. Rev. B **96**, 205407 (2017).

[14] J. Dufouleur, L. Veyrat, A. Teichgräber, S. Neuhaus, C. Nowka, S. Hampel, J. Cayssol, J. Schumann, B. Eichler, O. G. Schmidt, B. Büchner, and R. Giraud, Quasiballistic transport of Dirac fermions in a Bi_2Se_3 nanowire, Phys. Rev. Lett. **110**, 186806 (2013).

[15] J. Ziegler, R. Kozlovsky, C. Gorini, M.-H. Liu, S. Weishäupl, H. Maier, R. Fischer, D. A. Kozlov, Z. D. Kvon, N. Mikhailov, S. A. Dvoretsky, K. Richter, and D. Weiss, Probing spin helical surface states in topological HgTe nanowires, Phys. Rev. B **97**, 035157 (2018).

[16] D. Rosenbach, N. Oellers, A. R. Jalil, M. Mikulics, J. Kölzer, E. Zimmermann, G. Mussler, S. Bunte, D. Grützmacher, H. Lüth, and T. Schäpers, Quantum transport in topological surface states of selectively grown Bi_2Te_3 nanoribbons, Advanced Electronic Materials **6**, 2000205 (2020).

[17] S. Hikami, A. I. Larkin, and Y. Nagaoka, Spin-orbit interaction and magnetoresistance in the two dimensional random system, Progress of Theoretical Physics **63**, 707 (1980).
